# Overdot and overline annotation must be understood to accurately interpret $\dot{V}$ O_2MAX_ physiology with the Fick formula

**DOI:** 10.3389/fphys.2024.1359119

**Published:** 2024-02-20

**Authors:** Jayson R. Gifford, Christina Blackmon, Katelynn Hales, Lee J. Hinkle, Shay Richards

**Affiliations:** ^1^ Department of Exercise Sciences, Brigham Young University, Provo, UT, United States; ^2^ Program of Gerontology, Brigham Young University, Provo, UT, United States

**Keywords:** V.O_2MAX_, cardiac output, arteriovenous oxygen difference, endurance training, muscle oxygen diffusion

## Abstract

Few formulas have been used in exercise physiology as extensively as the Fick formula, which calculates the rate of oxygen consumption (*i.e.*, V.O_2_) as the product of cardiac output (Q.) and the difference in oxygen content in arterial and mixed venous blood (Δa $\bar{v}$ O_2_). Unfortunately, the physiology of maximum V.O_2_ (V.O_2MAX_) is often misinterpreted due to a lack of appreciation for the limitations represented by the oft-ignored superscript annotations in the Fick formula. The purpose of this perspective is to explain the meaning of the superscript annotations and highlight how such annotations influence proper interpretation of V.O_2MAX_ physiology with the Fick formula. First, we explain the significance of the overdots above V.O_2_ and Q., which indicate a measure per unit of time. As we will show, the presence of an overdot above Q. and lack of one above Δa $\bar{v}$ O_2_ denotes they are different types of ratios and should be interpreted in the context of one another—not in contrast to each other as is commonplace. Second, we discuss the significance of the overline above the “ $\bar{v}$ ” in Δa $\bar{v}$ O_2_, which indicates the venous sample is an average of blood that comes from mixed sources. The mixed nature of the venous sample has major implications for interpreting the influence of oxygen diffusion and blood flow heterogeneity on V.O_2MAX_. Ultimately, we give recommendations and insights for using the Fick formula to calculate V.O_2_ and interpret V.O_2MAX_ physiology.

## Introduction

In 1870, physiologist Adolph Fick reasoned one could apply the law of mass conservation to determine cardiac output (i.e., the volume of blood pumped by the heart per unit of time, Q.) by dividing the rate of systemic oxygen consumption (V.O_2_) by the simultaneously measured difference in oxygen content in arterial (C_a_O_2_) and mixed venous blood ( $C_{\bar{v}}O_{2}$ ) (Fick, 1870; Shapiro, 1972; Acierno, 1999). Since that time, the field of exercise physiology has relied heavily upon the equation derived from Fick’s original principle (Formula #1) and a simplified version in which the difference between C_a_O_2_ and $C_{\bar{v}}O_{2}$ is referred to as a single variable: the arterial-venous oxygen difference (Δa $\bar{v}$ O_2_; Formula #2). Δa $\bar{v}$ O_2_ is used to calculate V.O_2_ and make inferences regarding the extent to which central limitations (represented by Q) and peripheral factors (loosely represented by Δa $\bar{v}$ O_2_) affect one’s maximum rate of oxygen consumption (V.O_2MAX_).

$$\text{Formula }\#1:{˙VO}_{2}=˙Q\;x\;\left(C_{a}\;O_{2}\;-\;C_{\bar{v}}O_{2}\right)$$

$$\text{Formula }\#2\;\left(i.e.,\text{ Fick formula}\right):{˙VO}_{2}=˙Q\;x\;\Delta a\;\bar{v}\;O_{2}$$

Although the Fick formula has proven a valid and useful way to calculate V.O_2_ or Q., the assumptions and mathematical context of each variable must be appreciated to reach appropriate conclusions about the complex physiology of V.O_2MAX_. Unfortunately, the conditions and assumptions required by the Fick formula are often overlooked or oversimplified. These interpretations lead to incomplete or inappropriate conclusions about V.O_2MAX_ physiology. Many of the most-ignored conditions and assumptions of the Fick formula are avoided by understanding the oft-omitted overdot and overline annotations in it. Understanding and recognizing the meaning of these annotations will help physiologists make more appropriate conclusions about V.O_2MAX_ physiology. Therefore, we 1) highlight the conditions and assumptions of the Fick formula by explaining the meaning of the overdot and overline annotations and 2) encourage physiologists to consider these limitations when designing and interpreting studies regarding the physiology of V.O_2MAX_.

### How are variables in the Fick formula measured along the oxygen Cascade?

**FIGURE 1:**
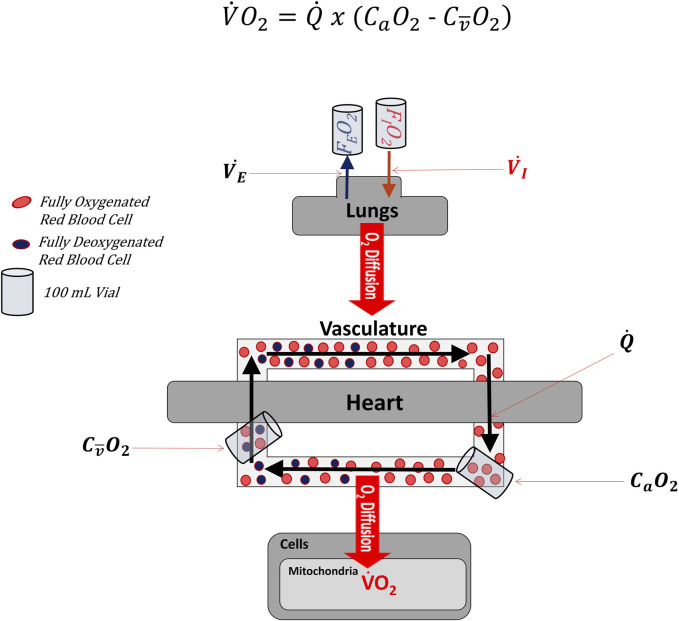
Simplified diagram lllustrating where variables found in the Fick Formula are measured along the cascade of oxygen from the atmosphere to mitochondria. Oxygen first enters the body through the lungs as a fraction of the inspired air (i.e., FIO_2_). Oxygen subsequently diffuses across the alveoli into the blood. The heart then pumps blood throughout the body. After leaving the heart, the vascular system constricts and dilates to direct oxygenated blood to active tissues. Once in the active tissues, some oxygen diffuses out of the blood into the distal cells (e.g., skeletal muscle), where it is consumed by the mitochondria in the process of oxidative phosphorylation. Partially deoxygenated blood leaves the active cells and returns to the heart and lungs, where it is ultimately reoxygenated. In the Fick Formula, cardiac output (Q.) represents the volume of blood leaving either side of the heart per minute. In the Fick Formula, arterial oxygen content (CaO_2_) is measured as the volume of oxygen contained within a fixed volume of arterial blood (typically 100 ml), while venous oxygen content (CO_2_) is measured as the volume of oxygen contained within a fixed volume of venous blood (typically 100 ml). Importantly, the CO_2_ used in the Fick Formula must represent the venous blood distal to the active tissues and as close to the heart as possible, so as to represent the average CO_2_ of the systemic circulation. FEO_2_: fraction of oxygen in expired air. V.I: Volume of inspired air per unit of time. V.E: Volume of expired air per unit of time.

Figure 1 illustrates multiple systems are involved in transporting oxygen from the ambient air to the mitochondria (Richardson, 2003; Wagner, 2008). Although oxygen consumption (V.O_2_) ultimately occurs within the many mitochondria of the body, total body V.O_2_ is most often measured by comparing the rate of oxygen inhalation and exhalation at the mouth via indirect calorimetry (Mtaweh et al., 2018).

Because indirect calorimetry often measures V.O_2_ at the mouth in an exercise physiology setting, one of the two remaining Fick formula variables (Q., Δa $\bar{v}$ O_2_) is usually also measured, and the other is derived from the Fick formula. Fick originally proposed Q. could be derived by dividing the V.O_2_ by the difference (signified by Δ in Δa $\bar{v}$ O_2_) between how much oxygen was in the blood leaving the heart (C_a_O_2_) and how much oxygen was in the blood returning to the heart ( $C_{\bar{v}}O_{2}$ ) (Acierno, 1999). To be valid, all measurements must be made simultaneously or during steady-state conditions. Figure 1 shows C_a_O_2_ is determined by measuring the amount of oxygen in a fixed volume (typically 100 mL or 1 dL) of arterial blood. The content of oxygen in arterial or venous blood is dependent upon the concentration of hemoglobin, its saturation with oxygen and the pressure of oxygen in plasma. The $C_{\bar{v}}O_{2}$ is determined by measuring the amount of oxygen in a fixed volume of venous blood sampled as close to the heart as possible where venous blood from the various tissues has mixed to create a venous sample representing the average of the *systemic circulation*. Cournand et al. (1943) determined the initial challenge of using the Fick formula to calculate Q. was the difficulty of obtaining *truly* mixed venous samples. If blood is drawn from the vascular system *before* all the blood is mixed (i.e., before all venous branches converge and mix their blood), the $C_{\bar{v}}O_{2}$ and Δa $\bar{v}$ O_2_ values will not represent what truly reaches the lungs, making subsequent calculations of Q. or Δa $\bar{v}$ O_2_ erroneous. Physiologists should also remember Δa $\bar{v}$ O_2_ is a difference between two values, and as such extraction is dependent upon C_a_O_2_ (*i.e.*, Δa $\bar{v}$ O_2_ cannot exceed C_a_O_2_).

Fick’s formula is often viewed as the gold standard of determining Q. (Hoeper et al., 1999). Nevertheless, exercise physiology labs often do not have the capability of sampling arterial or mixed venous blood, making the Fick-based approach of determining Q. less common in an exercise physiology setting. Alternative methods of estimating Q. have been developed: gas rebreathing, thermodilution, plethysmography and electrical bioimpedance are among those more commonly used in exercise physiology (Montero et al., 2015; Siebenmann et al., 2015). Importantly, each approach is associated with its limitations, shortcomings, and/or inherent errors (Siebenmann et al., 2015).

Some studies have applied the Fick formula to individual limbs or muscle groups to calculate the rate of oxygen consumption in specific regions of the body (Richardson et al., 1993; Boushel et al., 2011; Gifford et al., 2016). In cases of limb-specific V.O_2_, limb blood flow replaces Q. in the Fick formula and $C_{\bar{v}}O_{2}$ is measured in blood draining from the region of interest rather than venous blood in the central circulation.

### Why do $\dot{V}$ O_2_ and $\dot{Q}$ have overdots, but Δa $\bar{v}$ O_2_ does not?

The overdots in the Fick formula are often overlooked, misplaced, or forgotten, but they have important implications for interpreting V.O_2_ data. Dating back to Isaac Newton (this type of notation is often referred to as “Newton Notation”), “an overdot above a value indicates that the value “is a derivative taken with respect to time” (Weissten, 2019). Thus, the dot above V.O_2_ indicates it is a measure of the volume of oxygen consumed per unit of time. Cardiac output is denoted with an overdot (Q.) because it is the volume of blood ejected from a ventricle of the heart per unit of time (Brooks et al., 2005). In contrast, Δa $\bar{v}$ O_2_ is a measure of the *difference* in oxygen content per volume of blood rather than per unit of time and should not be expressed with an overdot.

One should not assume, however, because Δa $\bar{v}$ O_2_ has no unit of time that extraction occurs instantaneously. Indeed, oxygen extraction only occurs *while* the sampled blood is in the capillary, and, as indicated by the overdot above Q. (and emphasized by the multiplicative relationship denoted in Formula #2), that sampled blood is only in the capillary for a finite amount of time. The longer the sampled blood spends in the capillary, the more opportunity there is to extract oxygen.

If the extraction of oxygen is time sensitive, why is there no unit of time in Δa $\bar{v}$ O_2_? As illustrated in Formula #3 (Piiper and Scheid, 1981; Roca et al., 1992), units of time are present in multiple places in the equation for oxygen extraction:

$$\text{Formula }\#3:oxygen\;extraction=1-e^{\left(\frac{-DO_{2}}{\beta \times {\dot{Q}}_{cap}}\right)}$$

In this equation, DO_2_ is the diffusing capacity, measured in ml of oxygen diffused in a capillary per mmHg of pressure of O_2_ per unit of time (i.e., oxygen diffusion occurs over time). β is a coefficient derived from the slope of the oxyhemoglobin dissociation curve, and Q._cap_ is the volume of blood flowing through the capillary network per unit of time (Piiper and Scheid, 1981). The length of time which a volume of blood is near the extracting tissue (i.e., red blood cell transit time), which is influenced by Q._cap_ and Q., influences how much oxygen can be diffused or extracted (Angleys and Østergaard, 2020; Østergaard, 2020). Nevertheless, set up as a quotient, the time units in DO_2_ and Q._cap_ cancel each other out, making the final units of oxygen extraction or Δa $\bar{v}$ O_2_ have no reference to time. Experimental preparations which exclusively adjust Q._cap_ verify the clear inverse relationship between oxygen extraction and the rate of blood flow (Angleys and Østergaard, 2020). Understudied alterations in physiological function appear to reduce the negative impact of high flow on extraction *in vivo* (Richardson et al., 1993; Angleys and Østergaard, 2020).

### What does the overline above $\bar{v}$ indicate?

**FIGURE 2:**
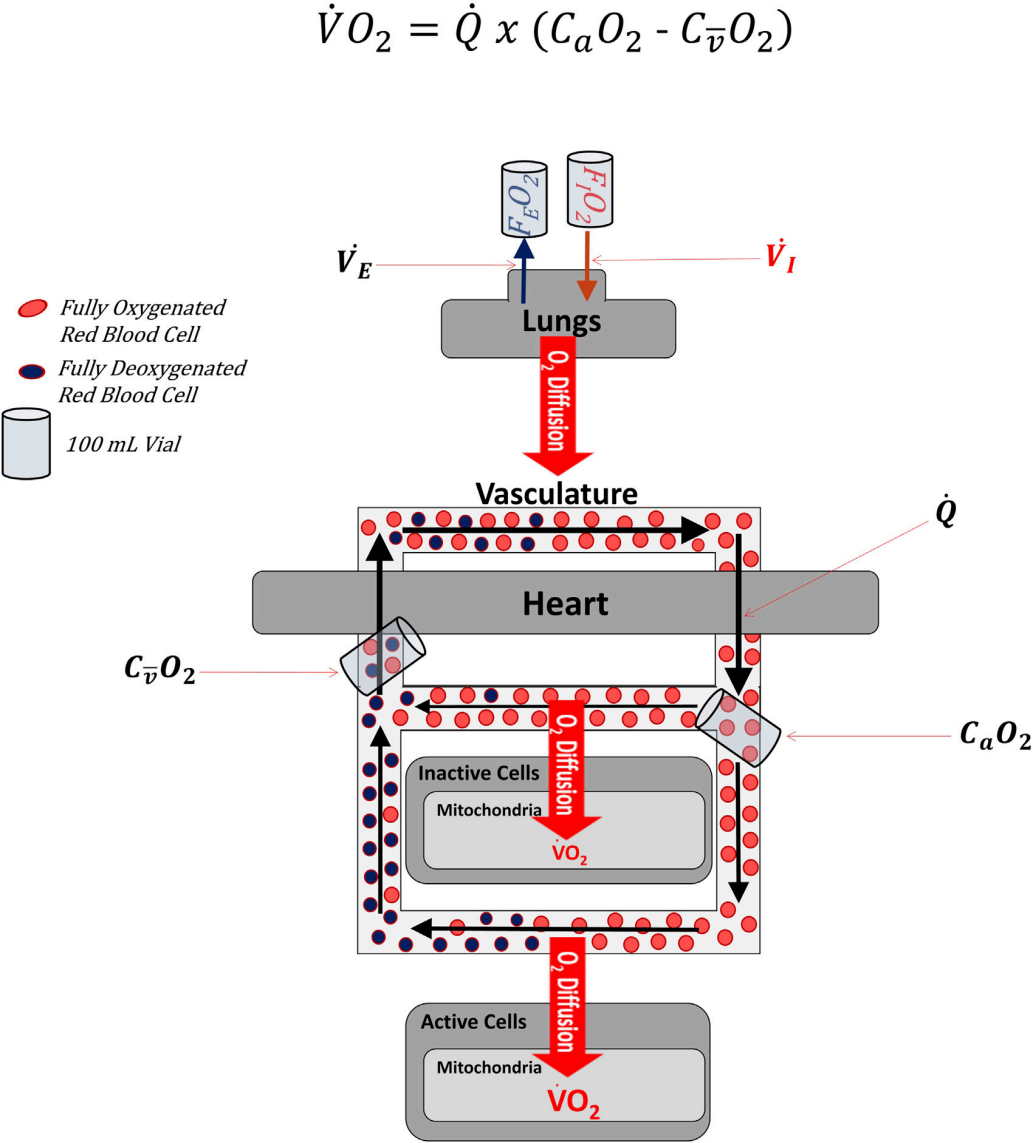
simplified diagram of the oxygen cascade illustrating that the venous sample used in the Fick Formula comes from mixed blood that represents the average of venous effluent from all regions of the body, whether active in the exercise or not. Note that the effluent leaving inactive cells is more oxygenated than the effluent leaving active cells. When the two effluents mix, the content of oxygen is no longer representative of the effluent of either the active or inactive cells, but of the average of the two.

Figure 1 illustrates the cardiopulmonary system as though all blood and oxygen went to the same place and experienced the same rates of flow and oxygen extraction. This is an oversimplification. Figure 2 depicts blood and oxygen being delivered to multiple regions of the body (simplified to just 2 different regions in Figure 2) with varying rates of flow and extraction. The overline above the $\bar{v}$ indicates that $C_{\bar{v}}O_{2}$ used to calculate Δa $\bar{v}$ O_2_ comes from a sample that represents the average venous oxygen content of the *entire body* (Weisstein, 2020), not just the active muscle. Indeed, even within an exercising muscle, blood flow distribution and oxygen consumption are heterogeneously distributed (Calbet et al., 2005; Heinonen et al., 2015). Different tissues have very different rates of blood flow, oxygen consumption (e.g., skin vs. skeletal muscle), and effluent $C_{\bar{v}}O_{2}$ . For the assumptions and calculations originally proposed by Fick to be accurate in a whole-body preparation, V.O_2_ and Q. must represent the whole body, and $C_{\bar{v}}O_{2}$ used to determine Δa $\bar{v}$ O_2_ must come from a mixed sample representing the average $C_{\bar{v}}O_{2}$ of the entire systemic circulation. Ultimately, the mixed nature of the venous effluent is true whether Δa $\bar{v}$ O_2_ is measured directly from blood or calculated as the ratio of whole-body V.O_2_ to Q.. As pointed out by a good reviewer, the overbar also highlights the assumption that the mixed sample is an average representation of all venous blood throughout the body. This assumption is generally met during steady-state exercise when flow rates and distributions are relatively steady. However, acute changes in regional blood flow distribution and rate, as are common during exercise transitions, may cause some regions to be temporarily over or underrepresented in that average. Thus, caution should be taken when interpreting the Fick Formula during non-steady state conditions.

## Discussion

Many physiologists have used the Fick formula to compartmentalize the factors leading to oxygen consumption into two general areas to discern what adaptations are meaningful to V.O_2MAX_ physiology. As discussed, the Fick formula has several assumptions, implied by overdot and overline notations, that are frequently overlooked when interpreting the Fick formula. Overlooking these implications may lead to erroneous or incomplete conclusions about factors affecting V.O_2MAX_. Interpreting previous and future data through the lens of assumptions may lead to an improved understanding of V.O_2MAX_ physiology and additional advancements in treatments for exercise intolerance.

### What the overdots imply about how to interpret changes in Δa $\bar{v}$ O_2_ and $\dot{Q}$

The overdot above Q. and lack of one above Δa $\bar{v}$ O_2_ should remind physiologists Δa $\bar{v}$ O_2_ is a different type of ratio than Q. and directly comparing changes in Δa $\bar{v}$ O_2_ to Q. lacks key insight. Δa $\bar{v}$ O_2_ is a volume percent, indicating the change in the volume of oxygen found within a volume of blood, and Q. is a volume per time (i.e., volume of blood ejected per minute). Importantly, blood is in motion (the denominator of Δa $\bar{v}$ O_2_), and only at a site for extraction for a limited time. In general, the greater the Q., the shorter the transit time of blood in the capillary (Kalliokoski et al., 2004), and the less time available for oxygen to be extracted.

If no other factors adjust, an increase in Q. will reduce the time during which O_2_ extraction can occur, thereby decreasing Δa $\bar{v}$ O_2_ (Angleys and Østergaard, 2020).

In contrast, if no other factors adjust, a decrease in Q. will provide a greater amount of time for extraction, resulting in a greater Δa $\bar{v}$ O_2_.

Clearly, the placement of the overdots in the Fick formula should remind physiologist that Δa $\bar{v}$ O_2_ is dependent upon Q. and must be interpreted in the context of Q., not in contrast to it.

### Interpreting Δa $\bar{v}$ O_2_ within the context of training-induced changes in $\dot{Q}$

Short-term endurance training typically increases V.O_2MAX_ and Q., while eliciting little-to-no change in Δa $\bar{v}$ O_2_ (Montero et al., 2015). Augmented blood volume, hemoglobin content and structural adaptations to the heart appear to facilitate the increase in Q. (Levine, 2008; Lundby and Montero, 2015). The lack of change in Δa $\bar{v}$ O_2_ is often interpreted as evidence that factors peripheral to the heart play little role in training-induced increase in V.O_2MAX_ (Levine, 2008; Montero et al., 2015). Unfortunately, *contrasting* the magnitude of change in Q. and Δa $\bar{v}$ O_2_ fails to consider the dependence of Δa $\bar{v}$ O_2_ upon Q.. Viewing Δa $\bar{v}$ O_2_ *through* the context of Q. indicates previously dismissed peripheral factors may play an important role in the training-induced increase in V.O_2MAX_.

For the sake of simplicity, suppose the heart in Figure 1 pumps 100 mL of blood containing 4 red blood cells (RBC) per second (i.e., Q. = 100 mL/s or 4 RBC/s) and the difference in the number of oxygenated RBC in the arterial and venous circulation (i.e., Δa $\bar{v}$ O_2_) is 2 RBC per 100 mL blood (i.e., extraction = 2 out of every 4 RBC). With 100 mL blood passing the periphery every second, the periphery deoxygenates blood at a rate of two RBC per second. Now suppose following endurance training, the Q. illustrated in Figure 1 doubled to 200 mL of blood per second (i.e., 8 RBC per second) and Δa $\bar{v}$ O_2_ remained constant at 2 RBC per 100 mL (i.e., extraction is still 2 out of every 4 RBC). To yield the same Δa $\bar{v}$ O_2_ (extraction rate), the periphery must have deoxygenated RBC *twice as fast* as before (*i.e.,* 4 RBC deoxygenated per second). If, as has been suggested at times (Montero et al., 2015), adaptations peripheral to the heart do not occur, or are not meaningful to the training-induced increase in V.O_2MAX_ (i.e., deoxygenation rate remained 2 RBC per second), the Δa $\bar{v}$ O_2_ would actually decrease to 1 RBC per 100 mL (i.e., 1 out of every 4 RBC) in the face of a doubled Q.. Interestingly, long-term training studies often report a training-induced *increase* in Δa $\bar{v}$ O_2_ (Montero et al., 2015), which *in the context of* Q., may indicate adaptations that facilitate extraction outpaced adaptations to Q..

The direct contrast of changes in Q. and Δa $\bar{v}$ O_2_ has guided the interpretation of the Fick formula for years, potentially leaving the conclusions of previous studies either incomplete or inaccurate. In a landmark study, Saltin et al. (1968) examined the impact of bedrest and endurance training on V.O_2MAX_. Observing equal changes in Δa $\bar{v}$ O_2_ and Q. with training, they concluded training-induced changes in V.O_2MAX_ were equally due to alterations in cardiac function and peripheral extraction. In 2015, Wagner (2015a) brought an updated perspective and interpreted the original Δa $\bar{v}$ O_2_ data from Saltin et al. within the context of simultaneous changes in Q., rather than in contrast to it. By interpreting Δa $\bar{v}$ O_2_ in the context of the increased Q., Wagner found evidence peripheral adaptations *outpaced* central adaptations and adaptations facilitating muscle oxygen extraction were *more* important to the observed increase in V.O_2MAX_ than were the observed changes in $\dot{Q}$ . In the case of Saltin et al., the importance of Δa $\bar{v}$ O_2_ was *understated*, not overlooked, although many studies reporting little-to-no change in Δa $\bar{v}$ O_2_ have concluded there was no Δa $\bar{v}$ O_2_ impact (Montero et al., 2015). Consequently, physiologists should carefully reconsider the conclusions of previous studies.

At this point relatively little is known about the training-induced adaptations that maintain extraction in the face of increased flow. Some suggest that training-induced increases in vascular function, increased capillary hematocrit, increased capillary density, and decreased flow heterogeneity (Poole et al., 2020) could potentially enhance diffusional conductance by increasing the area of the interface for diffusion. Meanwhile, evidence suggests training-induced increases in capillary density may be sufficient to slow capillary transit time in the face of increased Q. (Saltin, 1985; Kalliokoski et al., 2001). Still others contend that diffusional capacity is in excess to begin with, so adaptations are not necessary (see below). More research investigating the mechanisms responsible for the observed changes—or lack of changes—in Δa $\bar{v}$ O_2_ with training may lead to an improved understanding of V.O_2MAX_ physiology and may identify previously overlooked therapeutic targets for exercise intolerance.

### What the overline means for Fick-based interpretations of muscle oxygen diffusion

Oxygen must diffuse across the capillary into the muscle mitochondria to be used for oxidative phosphorylation. Nevertheless, even in cases of untapped mitochondrial respiratory capacity (Boushel et al., 2011; Gifford et al., 2016), oxygen is always found in the venous blood (Cardús et al., 1998; Boushel et al., 2011). When venous samples are drawn directly from veins draining a maximally exercising limb, approximately 15% of the oxygen that entered the limb through the arterial circulation is left in the venous blood (Richardson et al., 1993; Lundby and Montero, 2015). Some contend that limitations in muscle oxygen diffusion are the reason for the remaining oxygen in venous blood during maximal exercise (Wagner, 2015b), but others (Lundby and Montero, 2015) suggest this interpretation may ignore or underestimate the mixed nature of venous blood.

The overline above $\bar{v}$ should remind physiologists that $C_{\bar{v}}O_{2}$ comes from an average sample and is not representative of the venous oxygen content of any single region. Blood samples taken directly from veins that drain the region of interest (e.g., femoral vein for the quadriceps muscles (Gifford et al., 2016)) are less mixed than systemic samples, but these measures *remain* mixed samples: some of the venous blood is returning from less active regions of the exercising muscle or other less-active tissues (e.g., skin) (Heinonen et al., 2015). Unfortunately, the mixed nature of any venous sample makes it impossible to know with surety whether oxygen remaining in a venous sample is a result of impaired diffusion or whether the venous sample also contains blood from a less-active region.

Using a Fick-Wagner Diagram, several have provided compelling evidence that factors downstream of blood flow, usually identified as diffusional conductance, alter $C_{\bar{v}}O_{2}$ and contribute to commonly observed changes in V.O_2MAX_ in a variety of populations (Wagner, 2000; 2008; Hirai et al., 2015; Broxterman et al., 2020; Poole et al., 2020). However, readers must recognize, as the authors of such papers do (Piiper and Scheid, 1981; Roca et al., 1992; Wagner, 2015b; Lundby and Montero, 2015), the estimation of diffusional conductance inevitably comes with an asterisk, because it is reliant upon the assumption the venous sample comes exclusively from tissue that is homogenously active (Wagner, 2000; Boushel et al., 2011). The uncertainty about the origin of the venous sample makes it impossible to rule out the possibility observed changes in $C_{\bar{v}}O_{2}$ are due to changes in the precision of muscle blood flow (i.e., altered V.O_2_/Q. matching), rather than enhanced diffusional conductance (Lundby and Montero, 2015). Therefore, when considering data regarding muscle oxygen diffusion, physiologists must remember the mixed nature of the venous sample, represented by the overline in the Fick formula, or risk dismissing potentially meaningful adaptations and therapeutic targets that affect V.O_2_/Q. matching.

## Conclusion

Although the Fick formula is useful for measuring V.O_2_, the information it provides about the complex physiology of V.O_2MAX_ is often oversimplified, misinterpreted, and inaccurately stated. The overdot above Q. and the lack of one above Δa $\bar{v}$ O_2_ should remind physiologists these two variables have different units, which are dependent upon each other, and should be interpreted in the context of one another. Additionally, the overline above Δa $\bar{v}$ O_2_ should remind physiologists the venous sample used is an *average* of venous blood, and an average cannot capture the complex heterogeneity in blood flow and V.O_2_ distribution throughout the body. Appreciation for these annotations within context of each other and the Fick formula will help improve understanding of V.O_2MAX_ physiology and may help identify previously overlooked therapeutic targets for exercise intolerance.

## Data Availability

The original contributions presented in the study are included in the article/Supplementary material, further inquiries can be directed to the corresponding author.
